# Deaths Attributed to Respiratory Syncytial Virus in Young Children in High–Mortality Rate Settings: Report from Child Health and Mortality Prevention Surveillance (CHAMPS)

**DOI:** 10.1093/cid/ciab509

**Published:** 2021-09-02

**Authors:** Dianna M Blau, Vicky L Baillie, Toyah Els, Sana Mahtab, Portia Mutevedzi, Adama Mamby Keita, Karen L Kotloff, Ashka Mehta, Samba O Sow, Milagritos D Tapia, Beth A Tippett Barr, Benard O Oluoch, Clayton Onyango, Gunturu Revathi, Jennifer R Verani, Mahlet Abayneh, Nega Assefa, Lola Madrid, Joseph O Oundo, J Anthony G Scott, Quique Bassat, Inacio Mandomando, Antonio Sitoe, Marta Valente, Rosauro Varo, Ima-Abasi Bassey, Carrie Jo Cain, Amara Jambai, Ikechukwu Ogbuanu, Julius Ojulong, Muntasir Alam, Shams El Arifeen, Emily S Gurley, Afruna Rahman, Mustafizur Rahman, Jessica L Waller, Betsy Dewey, Robert F Breiman, Cynthia G Whitney, Shabir A Madhi, CHAMPS Consortium, Yasmin Adam, Janet Agaya, Sara Ajanovic, Addisu Alemu, Solomon Ali, George Aol, Henry Badji, Sanwarul Bari, Justina Bramugy, James Bunn, Richard Chawana, Atique Iqbal Chowdhury, Karen D Fairchild, Surafel Fentaw, Meerjady Sabrina Flora, Dickson Gethi, Nelesh P Govender, Carol L Greene, Tadesse Gure, Martin Hale, Juan Carlos Hurtado, Kitiezo Aggrey Igunza, Farzana Islam, J Kristie Johnson, Tatiana Keita, Sammy Khagayi, Iqbal Ansary Khan, Rima Koka, Diakaridia Kone, Nana Kourouma, Magdalene N Kuria, Sandra Lako, Sanjay G Lala, Hennie Lombaard, Ronita Luke, Thomas Misore, Paul K Mitei, Alexander M Ibrahim, Andrew Moultrie, Florence V Murila, Nellie Myburgh, Peter Nyamthimba, Richard Oliech, Richard Omore, Uma U Onwuchekwa, Stian M S Orlien, Louis Othieno, Peter Otieno, Kephas Otieno, Gregory Ouma, Benard Owuor, Shahana Parveen, Karen L Petersen, Mahbubur Rahman, Natalia Rakislova, Emily A Rogena, Doh Sanogo, Tahmina Shirin, Diakaridia Sidibe, Seydou Sissoko, Fatima Solomon, Gillian Sorour, James Sylvester Squire, Peter J Swart, Fikremelekot Temesgen, Sharon M Tennant, Bukiwe Nana Thwala, Cheick Bougadari Traore, Sithembiso Velaphi, Pio Vitorino, Jeannette Wadula, Melisachew Mulatu Yeshi

**Affiliations:** 1 Center for Global Health, Centers for Disease Control and Prevention, Atlanta, Georgia, USA; 2 South African Medical Research Council Vaccines and Infectious Diseases Analytics Research Unit, University of the Witwatersrand, Johannesburg, South Africa; 3 Centre pour le Développement des Vaccins, Ministère de la Santé, Bamako, Mali; 4 Center for Vaccine Development and Global Health, University of Maryland School of Medicine, Baltimore, Maryland, USA; 5 Centers for Disease Control and Prevention, Kenya, Kisumu, Kenya; 6 Kenya Medical Research Institute, Kisumu, Kenya; 7 Department of Pathology, Aga Khan University Hospital, Nairobi, Kenya; 8 National Center for Immunization and Respiratory Diseases, Centers for Disease Control and Prevention, Atlanta, Georgia, USA; 9 Division of Bacterial Diseases, National Center for Immunization and Respiratory Diseases, Centers for Disease Control and Prevention, Atlanta, Georgia, USA; 10 St Paul’s Hospital Millennium Medical College, Addis Ababa, Ethiopia; 11 College of Health and Medical Sciences, Haramaya University, Harar, Ethiopia; 12 Department of Infectious Disease Epidemiology, London School of Hygiene & Tropical Medicine, United Kingdom; 13 ISGlobal–Hospital Clínic, Unversitat de Barcelona, Barcelona, Spain; 14 Centro de Investigação em Saúde de Manhiça, Maputo, Mozambique; 15 Institutó Catalana de Recerca I Estudis Avançats (ICREA), Barcelona, Spain; 16 Pediatrics Department, Hospital Sant Joan de Déu, Universitat de Barcelona, Esplugues, Barcelona, Spain; 17 Consorcio de Investigación Biomédica en Red de Epidemiología y Salud Pública, Madrid, Spain; 18 Instituto Nacional de Saúde, Maputo, Mozambique; 19 ICAP–Columbia University, Makeni, Sierra Leone; 20 World Hope International, Makeni, Sierra Leone; 21 Ministry of Health and Sanitation, Freetown, Sierra Leone; 22 Crown Agents, Freetown, Sierra Leone; 23 International Center for Diarrhoeal Diseases Research (icddr,b), Dhaka, Bangladesh; 24 Johns Hopkins Bloomberg School of Public Health, Baltimore, Maryland, USA; 25 Emory Global Health Institute, Emory University, Atlanta, Georgia, USA

**Keywords:** Respiratory syncytial virus, child mortality, cause of death

## Abstract

**Background:**

Lower respiratory tract infections are a leading cause of death in young children, but few studies have collected the specimens needed to define the role of specific causes. The Child Health and Mortality Prevention Surveillance (CHAMPS) platform aims to investigate causes of death in children aged <5 years in high–mortality rate settings, using postmortem minimally invasive tissue sampling and other advanced diagnostic techniques. We examined findings for deaths identified in CHAMPS sites in 7 countries in sub-Saharan Africa and south Asia to evaluate the role of respiratory syncytial virus (RSV).

**Methods:**

We included deaths that occurred between December 2016 and December 2019. Panels determined causes of deaths by reviewing all available data including pathological results from minimally invasive tissue sampling, polymerase chain reaction screening for multiple infectious pathogens in lung tissue, nasopharyngeal swab, blood, and cerebrospinal fluid samples, clinical information from medical records, and verbal autopsies.

**Results:**

We evaluated 1213 deaths, including 695 in neonates (aged <28 days), 283 in infants (28 days to <12 months), and 235 in children (12–59 months). RSV was detected in postmortem specimens in 67 of 1213 deaths (5.5%); in 24 deaths (2.0% of total), RSV was determined to be a cause of death, and it contributed to 5 other deaths. Younger infants (28 days to <6 months of age) accounted for half of all deaths attributed to RSV; 6.5% of all deaths in younger infants were attributed to RSV. RSV was the underlying and only cause in 4 deaths; the remainder (n = 20) had a median of 2 (range, 1–5) other conditions in the causal chain. Birth defects (n = 8) and infections with other pathogens (n = 17) were common comorbid conditions.

**Conclusions:**

RSV is an important cause of child deaths, particularly in young infants. These findings add to the substantial body of literature calling for better treatment and prevention options for RSV in high–mortality rate settings.

Infections of the lower respiratory tract, such as pneumonia and bronchiolitis, are a leading cause of serious illness and death among children <5 years of age, with a disproportionate burden occurring in low- and middle-income settings [1]. Developing targeted treatment and prevention measures relies on understanding the burden and etiology of these infections. Studying etiology is difficult, however, because obtaining samples directly from the site of disease—inside the lung—is rarely possible. Studies of pneumonia etiology have relied primarily on tests of specimens from other sites, such as swab specimens of the upper respiratory tract or blood cultures, or specimens such as sputum that may contain oral flora, all of which pose significant limitations as proxies of what is really occurring in the lung; a limited number have included pleural fluid or lung aspirates.

Respiratory syncytial virus (RSV) is a common cause of lower respiratory tract infections (LRTIs), resulting in an estimated 3.2 million hospitalizations globally each year, with approximately 60 000 in-hospital deaths; just under half are among children <6 months of age [2]. The number of overall RSV-associated deaths may range between 55 000 and 200 000 annually [3]. However, meta-analytic estimates are limited by diagnostic assay variability and inconsistent case definitions among other considerations. Very limited primary data are available from which more precise, accurate estimates can be made [4].

The Pneumonia Etiology for Child Health (PERCH) study used a case-control design and mathematical modeling to determine the causes of LRTIs based on testing of blood samples, nasopharyngeal and oropharyngeal swab samples, induced sputum samples, and, in a small subset of cases, lung aspirates and pleural fluid samples. PERCH estimated that RSV accounted for 31% of chest radiography–confirmed pneumonia overall in children aged 1 to 59 months, with a range of 21%–36% among their different study sites in Sub-Saharan Africa and Asia, and suggested that detecting RSV in the upper respiratory tract of patients (when patients with lower respiratory tract infection were compared with controls) was highly associated with illness [5]. Similarly, RSV was found to be the leading pathogen in the Aetiology of Neonatal Infections in South Asia (ANISA) study [6]. In the Etiology of Pneumonia In the Community (EPIC) study, RSV was detected in 37% of pneumonia hospitalizations among children <5 years old in the United States, using similar diagnostic measures as the PERCH study [7].

Child and Mortality Prevention Surveillance (CHAMPS) was established to determine the causes of deaths among stillbirths, newborns, infants, and children <5 years of age in high–mortality rate settings. CHAMPS goes beyond previous work on pneumonia etiology by not only obtaining blood and upper respiratory tract samples, but also collecting multiple postmortem tissue samples from the lungs and other organs and examining them by polymerase chain reaction (PCR) and pathology techniques. These results, along with data from clinical records and verbal autopsy, are reviewed by trained multidisciplinary panels to attribute causes of death.

In this article, we focus on the role of RSV as a cause of child deaths. We describe how often RSV is identified among cases enrolled in CHAMPS and the characteristics of children who died of RSV. We examine circumstances when comorbidities along with RSV resulted in death and cases in which RSV was detected but not determined to be in the chain of events leading to death.

## METHODS

The analysis included deaths enrolled in CHAMPS sites between December 2016 and December 2019. CHAMPS protocol and methods are described elsewhere (www.champshealth.org) [8, 9]. Briefly, enrollment occurred in 10 sites in 7 countries in Asia and Africa: Baliakandi and Faridpur, Bangladesh; Harar and Kersa, Ethiopia; Siaya and Kisumu, Kenya; Bamako, Mali; Manhiça, Mozambique; Bombali, Sierra Leone; and Soweto, South Africa [10]. The CHAMPS site in Mozambique began enrollment in 2016, sites in South Africa, Kenya, Mali, and Bangladesh began in 2017, and Sierra Leone and Ethiopia began in 2019.

Children who were <5 years of age at the time of death and were residents of a catchment area were eligible for enrollment. Parents or guardians were approached for consent for verbal autopsy and clinical chart abstraction; permission was also sought for specimen collection and testing for children whose deaths were identified within 24 hours of occurrence. Only deaths occurring in children who were born alive and for whom complete specimen collection and testing were performed are included in this analysis. We did not include stillbirths because none enrolled in CHAMPS during this time period tested positive for RSV. Ethics committees at each site and at Emory University, Atlanta, Georgia, approved overall protocol and site ethics committees approved site specific protocols, respectively. The US Centers for Disease Control and Prevention committee deferred to the Emory University review.

CHAMPS pathology teams collected minimally invasive tissue samples [11] from enrolled subjects as well as peripheral blood, cerebrospinal fluid, stool or rectal swab, and nasopharyngeal swab samples. Site laboratories tested postmortem blood samples for human immunodeficiency virus DNA or RNA using PCR and for malaria using thick and thin smears and rapid diagnostic assays; blood and cerebrospinal fluid samples underwent microbial culture for bacterial pathogens. Real-time reverse-transcription PCR for detection of 116 pathogens was performed at each site using 4 custom-designed syndromic TaqMan array cards (TACs; Thermo Fisher Scientific) [12]; lung tissue and nasopharyngeal swab samples were tested for RSV and other respiratory pathogens, blood and cerebrospinal fluid samples for pathogens causing sepsis and meningitis, and stool samples for enteric pathogens. TAC testing targeted the RSV matrix (M) protein gene [12].

Tissue specimens were examined locally using routine histopathological techniques, and, if indicated by tissue appearance or TAC results, special staining techniques targeting microorganisms and immunohistochemistry were performed at the Infectious Diseases Pathology Branch of the Centers for Disease Control and Prevention [13]. Immunohistochemistry testing for RSV was performed on lung tissue using goat polyclonal antiserum raised against RSV.

Determination of cause of death (DeCoDe) panels convened at each site and reviewed information for each death once data collection and specimen testing were complete. The data reviewed included all postmortem diagnostics, clinical abstraction from child and maternal health records and verbal autopsy responses. DeCoDe panels consisted of ≥5 local experts, such as pediatricians, pathologists, obstetricians, nurses or other healthcare providers, epidemiologists, and microbiologists, with support from the CHAMPS Program Office [14]. Panels determined the chain of events leading to death using the World Health Organization (WHO) *International Statistical Classification of Diseases and Related Health Problems, Tenth Revision* (*ICD-10*) and WHO application of *ICD-10* for perinatal deaths (*ICD-PM*) guidelines [15,16].

For deaths in children attributed to a single condition, that condition was considered the underlying cause of death. For deaths in which >1 condition played a role, underlying, intermediate (also known as comorbid or antecedent), and immediate causes were assigned. We defined the causal chain or pathway to include all conditions listed as underlying, intermediate and immediate causes of death. Other factors that may have contributed but were deemed to not directly cause the death were listed as contributing (part 2 in the standard *ICD-10* death certificate) but not considered in the causal chain. To ensure consistency across DeCoDe panels, the network used diagnosis standards for defining common childhood causes of death, panel members underwent training, and a subset of cases were exchanged between sites for review. Panel members attributed deaths to pneumonia and/or RSV along with the certainty of that diagnosis based on predefined standards (Supplementary Table 1).

DeCoDe panels considered whether RSV belonged in the causal pathway of death, taking into account the role of concurrent medical conditions. Therefore, cases from which RSV was detected do not necessarily have RSV in the causal pathway. In addition to assigning conditions to the causal chain leading to death, DeCoDe panel members also considered whether appropriate management or prevention efforts could have prevented the death.

### Statistical Analysis

Data analyses examined features of deaths that were or were not attributed to RSV according to DeCoDe panel evaluations. Case studies were included to provide illustrative examples. Any death with RSV noted as an underlying, intermediate, or immediate cause was defined as an RSV death. LRTI deaths were defined as deaths in which pneumonia or viral pneumonitis was listed in the causal chain. We evaluated the data using descriptive analyses, such as frequency distributions, for 2 groups: (1) deaths in which RSV was determined to be in the causal chain by DeCoDe panel review and (2) deaths in which RSV was detected by PCR but for which DeCoDe panels determined that RSV did not cause the death. We conducted χ ^2^ analysis to compare differences between RSV deaths and deaths from other causes for factors such as age group (neonate, from birth to <28 days; infant, 28 days to <12 months; and child ,12 to <60 months), CHAMPS site, and other conditions present at death. Differences were considered statistically significant at *P* < .05. Analyses were conducted using SAS (version 9.4).

## RESULTS

**Figure 1.**
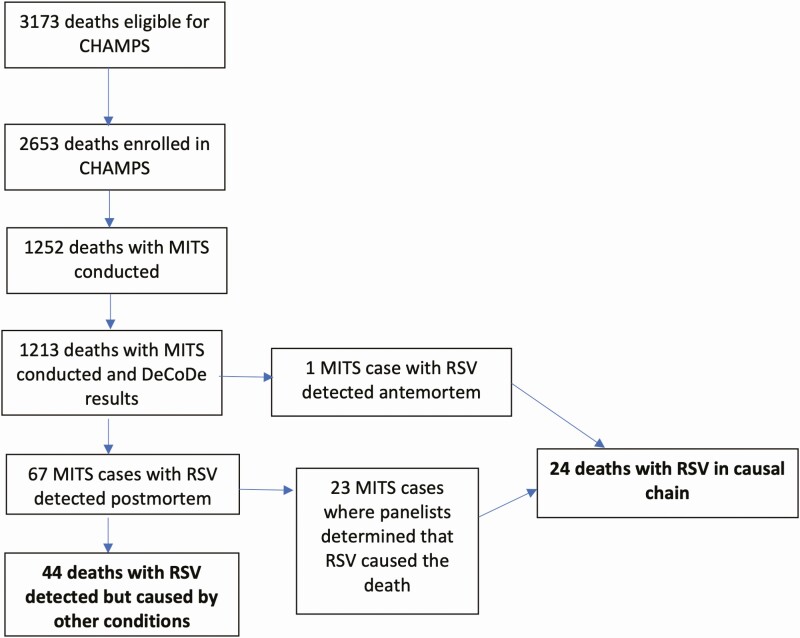
Flowchart of enrollment of deaths among children <5 years of age in Child Health and Mortality Prevention Surveillance (CHAMPS) sites, detection of respiratory syncytial virus (RSV) among deaths with minimally invasive tissue samples (MITS) tested for RSV, and identification of deaths determined by review panels (determination of cause of death [DeCoDe] panels) to be caused by RSV or other conditions.

Between December 2016 and December 2019, CHAMPS sites enrolled and determined causes for 1213 deaths in live-born children <5 years of age, including 695 (57%) deaths in neonates, 283 (23%) in infants, and 235 (19%) in children (Table 1). Among these deaths, RSV was detected in postmortem specimens in 67 (5.5% of all deaths), and it was determined to be a cause of death in 24 (2.0% of all deaths; Figure 1). RSV was the only or underlying cause for 4 (17%) deaths due to RSV and an immediate or antecedent cause for 20 (83%). RSV was determined to be a contributing rather than causal condition for 5 additional deaths. One death attributed to RSV as the underlying cause had RSV detected by premortem clinical testing. This death occurred in a 4-month-old infant who required intensive care for RSV bronchiolitis, was discharged and readmitted a week later with respiratory distress, and then died; the immediate cause of death was determined to be adenovirus pneumonia, and results of postmortem testing for RSV were negative.

**Table 1.** Characteristics of Neonatal, Infant, and Child Deaths Enrolled in Child Health and Mortality Prevention Surveillance (CHAMPS), for all Deaths Reviewed by Determination of Cause of Death Panels, Deaths Those With Respiratory Syncytial Virus (RSV) Detected at Postmortem Testing, and Deaths With RSV in the Causal Chain

Characteristic	Deaths, No. (%)^a^		
	All Deaths (N = 1213)	Deaths With RSV Detected (n = 67)	Deaths With RSV in Causal Chain (n = 24)
Age at death			
<24 h	289 (24)	5 (7.5)	0 (0)
Early neonate (24 h to 6 d)	269 (22)	2 (3.0)	0 (0)
Late neonate (7 to 27 d)	137 (11)	6 (9.0)	3 (12)
Infant (28 d to <6 mo)	184 (15)	22 (33)	12 (50)
Infant (6 to <12 mo)	99 (8.0)	11 (16)	3 (12)
Child (12 to <60 mo)	235 (19)	21 (31)	6 (25)
Age at death, mean (median), d	200 (10)	339 (166)	276 (139)
Male sex	677 (56)	32 (48)	10 (42)
Location of death			
Community^b^	194 (16)	16 (24)	5 (21)
Health facility	1019 (84)	51 (76)	19 (79)
Season of death			
Peak respiratory season^c^	602 (50)	52 (78)	19 (79)
Not respiratory season	611 (50)	15 (22)	5 (21)
Time between death and MITS procedure, median (range), h	15 (0–97)	16 (0–63)	19 (5–47)
CHAMPS site			
Bangladesh	80 (7)	2 (3.0)	0 (0)
Ethiopia	28 (2)	3 (4.5)	1 (4.2)
Kenya	281 (23)	18 (27)	4 (17)
Mali	103 (8)	10 (15)	4 (17)
Mozambique	125 (10)	3 (4.5)	0 (0)
Sierra Leone	99 (8)	2 (3.0)	0 (0)
South Africa	497 (41)	29 (43)	15 (63)

Abbreviations: CHAMPS, Child Health and Mortality Prevention Surveillance; MITS, minimally invasive tissue sampling; RSV, respiratory syncytial virus.

^a^Data represent no. (%) of deaths unless otherwise specified.

^b^Community deaths include those that occurred at home or on the way to a health facility.

^c^Respiratory season was defined as follows for the surveillance sites: Bangladesh, June–September; Ethiopia, April–May and October–November; Kenya, May–September; Mali, July–September; Mozambique, January–July; Sierra Leone, September–January; and South Africa, February–September.

**Figure 2.**
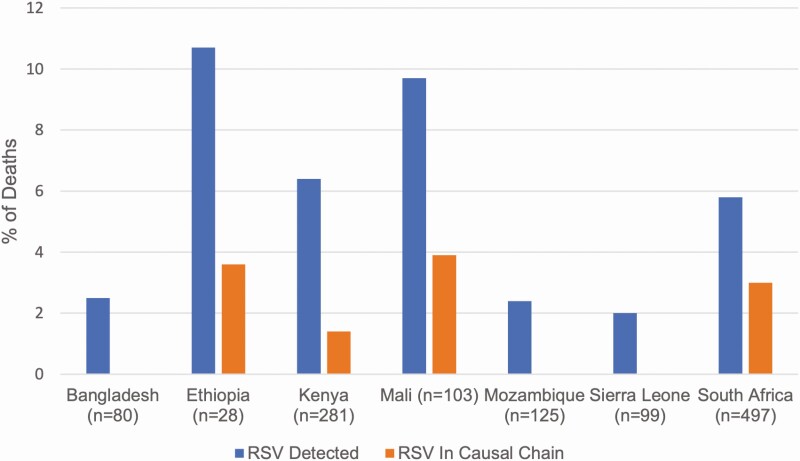
Proportion of deaths with respiratory syncytial virus (RSV) detected and with RSV determined to be in the causal chain leading to death, by Child Health and Mortality Prevention Surveillance (CHAMPS) site.

Most deaths with RSV detected were identified in the South Africa CHAMPS site (29 of 67 [43.3%]), which represents a similar proportion of total deaths enrolled in CHAMPS during this time (497 of 1213 [41.0%]). Proportionally, RSV was detected most frequently in cases from Ethiopia (3 of 28 [10.7%]) and Mali (10 of 103 [9.7%]) and listed in the causal chain most frequently in the same countries (3.6% and 3.9%, respectively (Figure 2 and Supplementary Table 2). RSV was rarely detected among deaths occurring within the first week of life (7 of 558 [1.3%]); none of the deaths in this age group were attributed to RSV or determined to have RSV as a contributing condition (Figure 2). RSV was detected among 11.7% (33 of 283) of deaths in infants (aged 28 days to <12 months) and in 8.9% (21 of 235) of children.

Younger infants (aged 28 days to <6 months of age) accounted for half of all deaths attributed to RSV (12 of 24 [50%]), and RSV was determined to have caused 6.5% of all deaths in this age group. Among all cases with RSV detected, younger infants more commonly had RSV determined to be a cause of death when detected (12 of 22 [54.5%]) compared with older infants and children (9 of 32 [28.1%]; *P* = .05). Infants <4 months of age, the age group most likely to be protected by maternal RSV immunization, accounted for 8 of 24 deaths caused by RSV (33%) and 18 of 67 RSV detections (27%). Deaths with RSV detection occurring during the typical respiratory season at each site were more often attributed to RSV (19 of 602 [3.2%]) than deaths in other seasons (5 of 611 [0.8%]; *P* = .003). Similarly, RSV was detected more often during the respiratory season for each site (52 of 602 [8.3%]) than during other seasons (15 of 611 [2.5%]; *P* < .001). Although this difference was not significant, a higher proportion of community deaths (those occurring outside a health facility) had RSV detected, when compared with the deaths within a health facility (8.3% vs 4.9%, respectively).

All deaths with lung tissue that tested positive by immunohistochemistry for RSV (10 of 19 tested with immunohistochemistry) had RSV in the causal chain. Likewise, RSV was determined to be in the causal chain more often for deaths in which RSV was detected by PCR in postmortem samples from both the nasopharynx and lungs (17 of 22 [77.3%]; Table 2), compared with deaths in which RSV was detected in only 1 specimen type (4 of 31 [12.9%] with only the nasopharyngeal swab sample positive and 2 of 14 [14.3%] with only lung tissue positive; both *P* < .001). None of the deaths within the first 7 days of life had RSV detected by PCR in both specimen types (Table 2).

**Table 2.** Postmortem Test Results and Attribution of Respiratory Syncytial Virus as Cause of Death by Child Health and Mortality Prevention Surveillance (CHAMPS) Panel, by Postmortem Specimen Type and Age Group

Age Group	Specimens Tested, No.	Deaths with RSV in Causal Chain/Deaths with specimens testing positive for RSV, No. (%)			
		Only NP Swab Sample Positive	Only Lung Tissue Positive	Both NP Swab Sample and Lung Tissue Positive	Total^a^
<24 h	289	0/3	0/2	0/0	0/5
Early neonate (1–6 d)	269	0/1	0/1	0/0	0/2
Late neonate (7–27 d)	137	0/1	0/2	3/3 (100)	3/6 (50)
Infant (28 d to <6 mo)	184	0/8	2/5 (40)	9/9 (100)	11/22 (50)
Infant (6 to <12 mo)	99	2/6 (33.3)	0/3	1/ 2 (50)	3/11 (27)
Child (12–59 mo)	235	2/12 (16.7)	0/1	4/8 (50)	6/21 (29)
Total	1213	4/31 (12.9)	2/14 (14.3)	17/22 (77.3)	23/67 (34)

Abbreviations: NP, nasopharyngeal; RSV, respiratory syncytial virus.

^a^One death among our identified RSV deaths was determined to be caused by RSV based on antemortem testing and is not included in this table.

**Figure 3.**
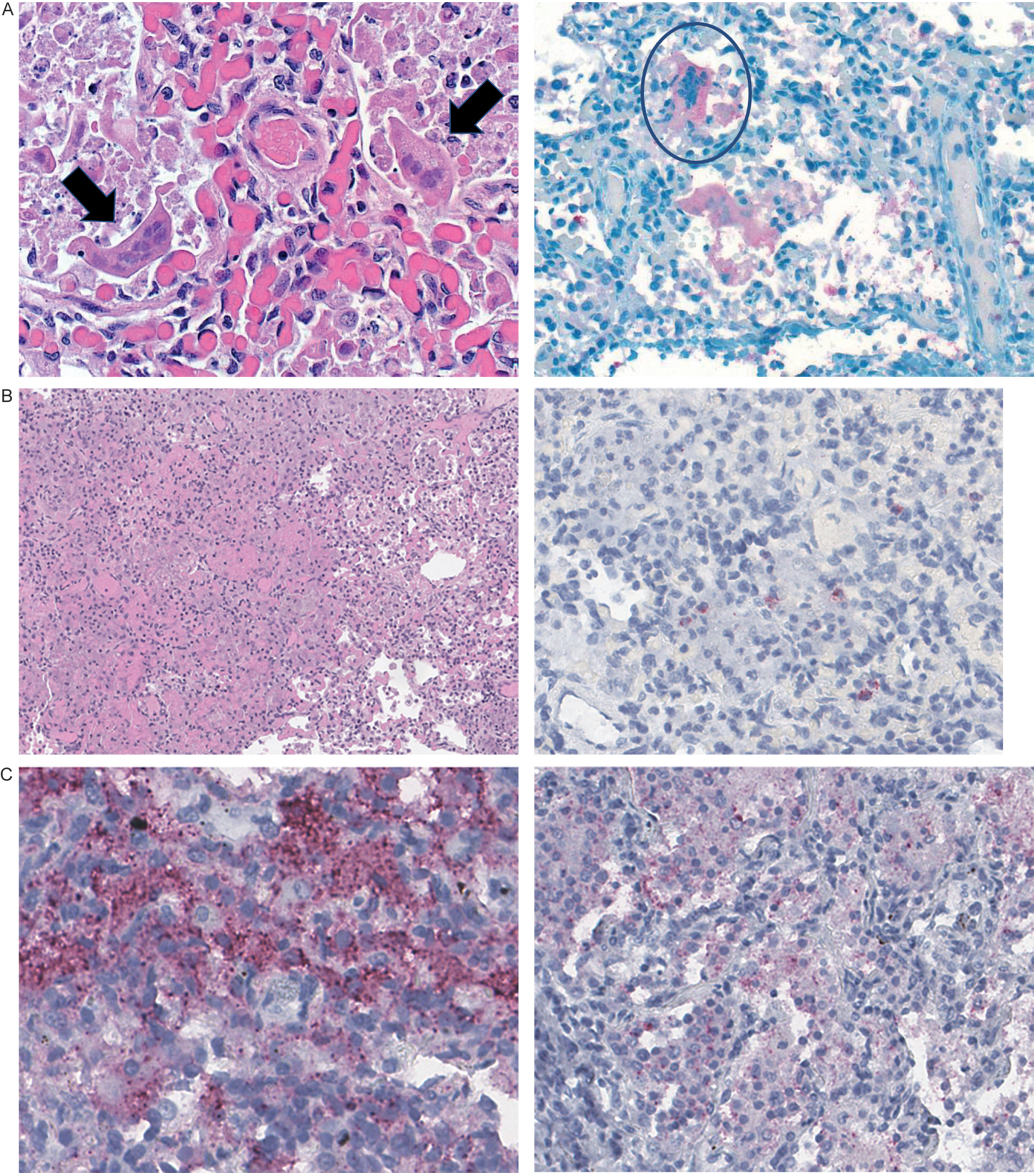
*A.* Findings in a newborn with respiratory syncytial virus (RSV) determined to be the underlying and only cause of death. The subject was a 22-day-old girl, born in a healthcare facility at 38 weeks’ gestation (birthweight, 3400 g) to a mother who was positive for human immunodeficiency virus (HIV) and receiving treatment. The newborn was seen at a referral hospital after a week of difficulty breathing; the father refused admission, and the newborn was discharged on antibiotics. She developed a fever, was seen in the clinic, and was again referred for hospital care. She died at home that night. Postmortem testing was negative for HIV, tuberculosis, and malaria; blood and cerebrospinal fluid cultures were also negative. Polymerase chain reaction (PCR) testing identified RSV in lung tissue and a nasopharyngeal (NP) specimen; the latter also contained *Klebsiella pneumoniae, Staphylococcus aureus, Moraxella catarrhalis, Haemophilus influenzae,* and rhinovirus. Pathology found mild steatosis and extramedullary hematopoiesis in the liver, and interstitial pneumonitis in specimens from both lungs. Both images show lung tissue; the arrows in left image indicate multinucleated syncytial cells, and the circle in right image, positive immunohistochemical (IHC) staining in these cells. *B,* Findings after death caused by a chronic infected ulcer (underlying cause), with subsequent pneumonia caused by *S. aureus* and *Streptococcus pneumoniae* (morbid causes) and *S. aureus* sepsis (immediate cause) in a malnourished infant (part 2 contributing cause); RSV was detected but not deemed a cause of death. This subject was a 6-month-old girl born at 36 weeks’ gestation (birthweight, 3165 g). She presented with 1 day of cough, fever, lethargy, and decreased milk intake and 2 days of diarrhea. She had been receiving oral and topical medications for an occipital ulcer. At the admission examination, she was hot to the touch, hydrated, and in respiratory distress, with grunting, alar flaring, a respiratory rate of 62/min, and a pulse rate of 178 /min. She was treated with gentamicin and ampicillin but died the following day. *S. aureus* grew in cultures collected before death from the occipital abscess and blood. The infant’s weight and height at postmortem examination were 6.07 kg and 60 cm, respectively (weight-for-age *z* score, −2.18; weight-for-height *z* score, −1.5). Results of postmortem tests for HIV, tuberculosis, and malaria were negative. *S. aureus* grew in lung tissue culture. PCR testing detected *S. aureus* in blood and lung tissue. Lung tissue was also positive for *S. pneumoniae*. PCR results in an NP swab specimen were positive for RSV, *H. influenzae, M. catarrhalis, S. pneumoniae,* and *S. aureus*; a stool sample was positive for adenovirus 40/41 and *Escherichia coli*. Liver tissue pathology showed severe large- and small-droplet steatosis. Right and left lung specimens showed severe necrotizing bronchopneumonia (*left image*), with IHC staining positive for *S. aureus* (not shown) *and S pneumoniae* (*red staining in right image*). *C,* Findings after death in a child caused by HIV with wasting syndrome (underlying cause), malaria (morbid condition), *and E. coli* sepsis (immediate cause); severe anemia and polymicrobial pneumonia due to RSV, *E. coli*, and *S. pneumoniae* were considered contributing (part 2) causes. The child was a 21-month-old girl admitted to the hospital with a history of cough, fever, vomiting, and rash localized at her mouth, elbows, and buttocks. On examination, she appeared ill and wasted and was febrile. Antemortem diagnoses included severe anemia, severe malaria, pneumonia, and malnutrition; she died on hospital day 11. Postmortem weight and height were 6.7 kg and 82.1 cm, respectively (weight-for-age *z* score, −4.04; weight-for-height *z* score, −5.1). At postmortem examination, she appeared malnourished and pale, with healed sacral and trochanteric pressure sores and right elbow sores. HIV testing results were positive; results of testing for tuberculosis and malaria were negative, and a blood culture grew *E. coli*. PCR testing of blood identified *E. coli and Plasmodium falciparum*; lung tissues were positive for RSV, cytomegalovirus, *M. catarrhalis, S. pneumoniae, Pneumocystis jirovecii, and K. pneumoniae*; an NP specimen was positive for RSV, *K. pneumoniae, S. pneumoniae, S. aureus, M. catarrhalis, Pseudomonas aeruginosa,* and cytomegalovirus; and a stool sample was positive for *E. coli*, and *Enterovirus*. At pathological examination of the liver, extensive large- and small-droplet steatosis and sinusoidal leukocytosis were noted, and results of IHC testing were positive for malaria; bacterial bronchopneumonia and focal interstitial pneumonitis were seen in lung tissue. IHC tests in lung tissuee detected *E. coli* (*left image*) and *S. pneumoniae* (*right image*).

**Figure 4.**
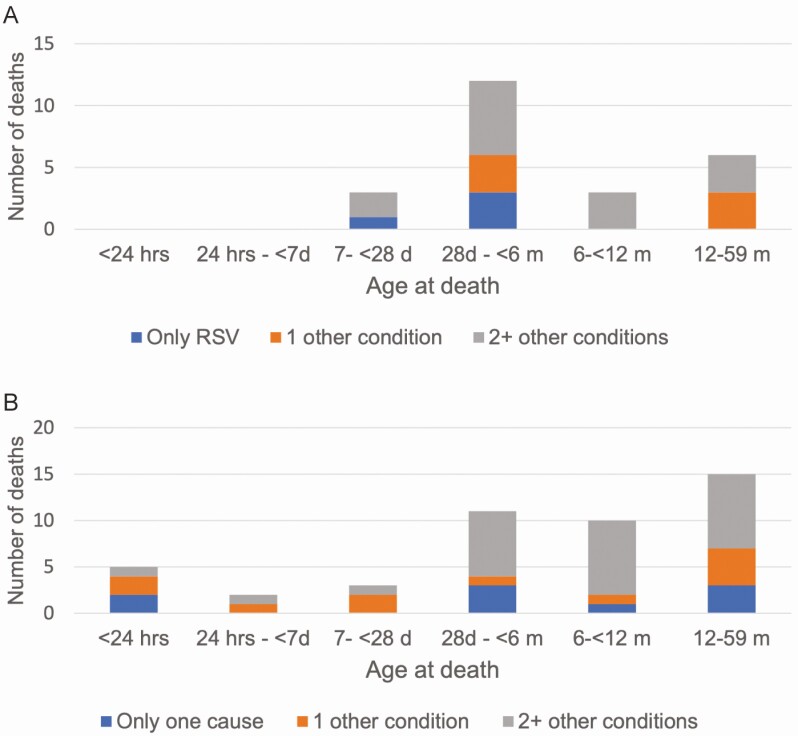
A. Number of conditions in the causal chain for deaths attributed to respiratory syncytial virus (RSV) (n = 24), by age group. *B,* Deaths in which RSV was detected but not determined to be in the causal pathway (n = 43).

**Figure 5.**
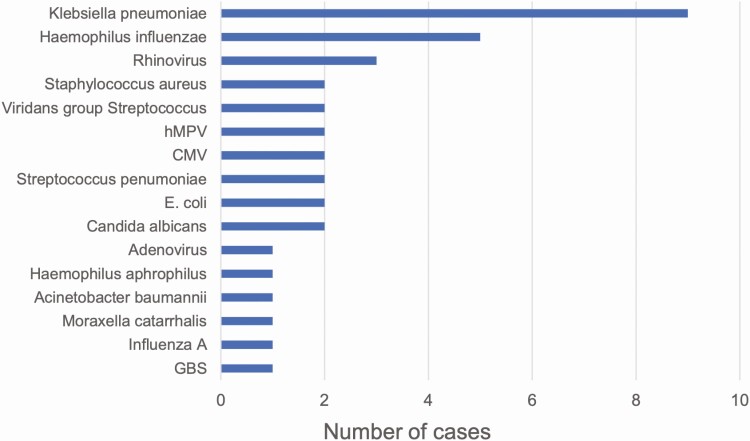
Other pathogens determined to be in the causal chain leading to death. Abbreviations: CMV, cytomegalovirus; E coli, *Eschericia coli*; GBS, Group B streptococcus; hMPV, human metapneumovirus.

Among the 24 deaths in which RSV was in the causal chain, RSV was determined to be the underlying and only cause of death for 4 cases (16.7%; example in Figure 3B). Among those with other conditions, the median number of other conditions in the causal chain was 2 (range, 1–5; Figure 4A). The number of other conditions in the causal chain did not differ between deaths before 6 months of age and deaths among older children (both with a median of 2 other conditions) or between whose deaths occurring in the community and those occurring in health facilities (both median 2 other conditions). Birth defects were common among deaths attributed to RSV, being listed in the causal chain for 8 (33.3%) deaths (Supplementary Table 3). Seventeen of 24 RSV-associated deaths (70.8%) had LRTIs attributed to another pathogen in the causal pathway in addition to RSV; *Klebsiella pneumoniae* (n = 9), *Haemophilus influenzae* (n = 4), and rhinovirus (n = 3) were most common among the other pathogens identified in these deaths (Figure 5).

**Figure 6.**
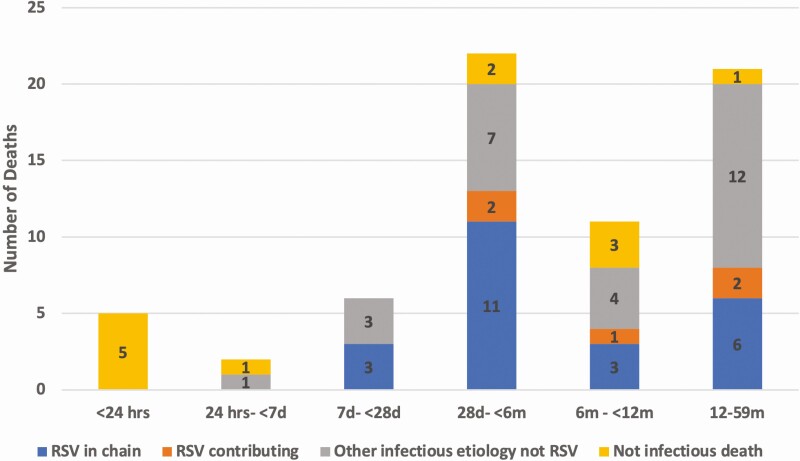
Causes of death determined by Child Health and Mortality Prevention Surveillance (CHAMPS) review (determination of cause of death) panels for the 67 deaths with specimens that tested positive for respiratory syncytial virus (RSV), by age group. Results are grouped by whether (1) RSV was in the causal chain of events leading to death (*blue*); (2) RSV contributed, but other conditions caused the death (*orange*); (3) infections other than RSV caused the death (*gray*); or (4) the death was caused by conditions that were not infectious.

DeCoDe panels determined that other infectious causes caused death in the majority (27 of 39 [69.2%]) of deaths in children <5 years of age with RSV detected in postmortem specimens but for which RSV was not a causal or contributing condition (Figure 6; examples in Figures 3B and 3C). The median number of conditions in the causal chain in this group was 2 (range, 1–6; Figure 4B); 21 of 39 deaths (54%) did not have an LRTI of any type among the conditions listed. Among 7 deaths that occurred in the first week of life with RSV detected, 1 was attributed to another infection (pneumonia due to *K. pneumoniae*), and 6 were determined to be caused by noninfectious causes, including complications of preterm birth (n = 2), birth defects (n = 2), hypoxic ischemic encephalopathy (n = 1), and perinatal asphyxia (n = 1).

## Discussion

Our results confirm that RSV is an important cause of death in children <5 years old, especially among infants 28 days to <6 months of age; in this age group, 1 of every 15 deaths was caused by RSV. Half of the deaths identified and attributed to RSV were in these younger infants, as were cases in which RSV was thought to have contributed to, but not caused, the death. The findings on age distribution and seasonality for RSV are consistent with previous work describing RSV burden and epidemiology [17].

DeCoDe panelists more often attributed a death to RSV when immunohistochemical staining identified RSV in lung tissue or when PCR demonstrated RSV in both lung tissue and nasopharyngeal specimens. Just under half of the deaths in which RSV was detected were attributed to RSV by CHAMPS DeCoDe panels. About 1 in 4 deaths with RSV detected that were ultimately not determined to be RSV deaths were not attributed to any infectious cause, suggesting that RSV may have been present but not causing serious symptoms in these children. Our findings differ from studies of RSV prevalence among healthy children. In the multicountry PERCH study, only about 4% of control children <5 years of age in the community had RSV detected in upper airway samples [5], and in the US EPIC study, <3% of asymptomatic control children had RSV detected [7]. While CHAMPS methods include careful evaluation of lung tissue with sensitive techniques, such testing can miss part of the sequence of events leading to death, and some deaths not attributed to RSV based on postmortem findings may have been attributed to RSV if diagnostic test results had been available from earlier in the illness course. For example, the *Staphylococcus aureus* pneumonia death described in Figure 3B may have been the result of an earlier RSV infection in which the virus was no longer detectable in the lung at time of death, but the DeCoDe panel could not make such a determination from the available evidence.

RSV causes many serious infections in healthy infants and young children. A large majority of the deaths we identified and attributed to RSV, however, were in children who had other conditions in the causal chain that led to their deaths. This suggests that RSV deaths mostly occur among children who are especially vulnerable, such as those with a birth defect or concurrent bacterial infections or whose RSV leads to other subsequent infections with other pathogens. The number of different pathogens identified and determined to be in the causal chain highlights the fact that severe pneumonia episodes may often be caused by >1 pathogen. In addition, our results demonstrate the hazard of attributing causality to antemortem diagnostic testing results, in particular those from the upper respiratory tract, or to microbiological results in the absence of histopathological evidence.

Applying histopathology to investigations of cause of death allows a substantial capacity to discern causes of severe illnesses leading to death that is much more specific and robust than methods more routinely used in high–mortality rate settings, such as verbal autopsy [18]. Nonetheless, our study has important limitations. First, CHAMPS is relatively new in some sites, meaning that a longer duration of surveillance might detect years in which RSV is more prevalent than observed to date. Next, RSV could cause infections that lead to fatal complications, but the pathogen may be undetectable in postmortem samples at the time of death, especially if the death occurred many days or even weeks after illness onset. In addition, in deaths that occur in children with complicated medical courses, discerning the role of RSV among many possible causes can be difficult. Finally, the number of RSV-associated deaths occurring in the community may be underappreciated by the results presented here, as thus far CHAMPS more often enrolls deaths that occur in health facilities. Taken together, these challenges suggest that the proportion of deaths in infants and children attributed to RSV in this analysis should be considered a minimal estimate of the true burden. Linking CHAMPS data to other data on RSV infections is needed to better quantify the burden of RSV and its role in deaths.

Our findings join the substantial body of literature calling for better treatment and prevention measures for RSV, in particular for high–mortality rate settings. While review of the treatments received by the children in our study was beyond the scope of this analysis, improving the availability of oxygen and other types of supportive care in hospitals in low- and middle-income countries would likely improve outcome for many patients with RSV. It is a WHO priority to develop RSV monoclonal antibodies and vaccines that could be given to infants or vaccines that mothers would receive during pregnancy [19], and a recent review identified dozens of products in development or clinical trials [20, 21].

## Supplementary Data

Supplementary materials are available at *Clinical Infectious Diseases* online. Consisting of data provided by the authors to benefit the reader, the posted materials are not copyedited and are the sole responsibility of the authors, so questions or comments should be addressed to the corresponding author.

ciab509_suppl_Supplementary_Tables

## Notes

***Acknowledgments.*** CHAMPS extends its deepest thanks to all the families who participated in this study.

***Financial support*.** CHAMPS is supported by the Bill & Melinda Gates Foundation (grant OPP1126780).

***Supplement sponsorship.*** This supplement is sponsored by the Bill and Melinda Gates Foundation.

***Potential conflicts of interest*.** K. L. K. reports grants from Emory University during the conduct of the study. A. M. reports grants from Emory University during the conduct of the study. S. O. S reports grants from Emory University and grants from the University of Maryland, Baltimore, during the conduct of the study. M. D. T. reports grants from Emory University, for the work presented here, during the conduct of the study. J. A. G. S. reports grants from Emory University during the conduct of the study and has received grants from Wellcome Trust, the Medical Research Council, the National Institute for Health Research, the Bill & Melinda Gates Foundation, and Gavi, outside of the submitted work. All other authors report no potential conflicts. All authors have submitted the ICMJE Form for Disclosure of Potential Conflicts of Interest. Conflicts that the editors consider relevant to the content of the manuscript have been disclosed.

***CHAMPS Consortium.*** The CHAMPS Consortium includes the following: Yasmin Adam (University of the Witwatersrand and the Department of Obstetrics & Gynaecology, Chris Hani Baragwanath Academic Hospital), Janet Agaya (Kenya Medical Research Institute, Kisumu), Sara Ajanovic (ISGlobal–Hospital Clínic, Unversitat de Barcelona, Spain), Addisu Alemu (College of Health and Medical Sciences, Haramaya University, Harar, Ethiopia), Solomon Ali (Ethiopian Public Health Institute, Addis Ababa, Ethiopia), George Aol (Kenya Medical Research Institute, Kisumu), Henry Badji (Center for Vaccine Development and Global Health, University of Maryland School of Medicine, Baltimore), Sanwarul Bari (International Center for Diarrhoeal Diseases Research [icddr,b], Dhaka, Bangladesh), Justina Bramugy (Centro de Investigação em Saúde de Manhiça, Maputo, Mozambique), James Bunn (World Health Organization–Sierra Leone Office, Freetown), Richard Chawana (University of Witwatersrand and MRC Vaccines and Infectious Diseases Analytics Research Unit, Johannesburg, South Africa), Atique Iqbal Chowdhury (International Center for Diarrhoeal Diseases Research [icddr,b], Dhaka, Bangladesh), Karen D Fairchild (Pediatrics Department, University of Virginia, Charlottesville), Surafel Fentaw (Ethiopian Public Health Institute, Addis Ababa, Ethiopia), Meerjady Sabrina Flora (Institute of Epidemiology, Disease Control, and Research, Dhaka, Bangladesh), Dickson Gethi (Kenya Medical Research Institute, Kisumu), Nelesh P Govender (University of Witwatersrand and National Institute for Communicable Diseases, Johannesburg, South Africa), Carol L Greene (Center for Vaccine Development and Global Health, University of Maryland School of Medicine, Baltimore), Tadesse Gure (College of Health and Medical Sciences, Haramaya University, Harar, Ethiopia), Martin Hale (University of Witwatersrand, and National Health Laboratory Service, Johannesburg, South Africa), Juan Carlos Hurtado (ISGlobal–Hospital Clínic, Unversitat de Barcelona, Spain), Kitiezo Aggrey Igunza (Kenya Medical Research Institute, Kisumu), Farzana Islam (International Center for Diarrhoeal Diseases Research [icddr,b], Dhaka, Bangladesh), J Kristie Johnson (Center for Vaccine Development and Global Health, University of Maryland School of Medicine, Baltimore), Tatiana Keita (Clinique Pasteur, Bamako, Mali), Sammy Khagayi (Kenya Medical Research Institute, Kisumu), Iqbal Ansary Khan (Institute of Epidemiology, Disease Control, and Research, Dhaka, Bangladesh), Rima Koka (Center for Vaccine Development and Global Health, University of Maryland School of Medicine, Baltimore), Diakaridia Kone (CSRef Commune I, Bamako, Mali), Nana Kourouma (Centre pour le Développement des Vaccins, Ministère de la Santé, Bamako, Mali), Magdalene N Kuria (Kisumu East District Hospital, Kenya), Sandra Lako (Aberdeen Women’s Centre, Freetown, Sierra Leone), Sanjay G Lala (University of Witwatersrand, Johannesburg, and Department of Pediatrics and Child Health, Chris Hani Baragwanath Academic Hospital, Soweto, South Africa), Hennie Lombaard (University of Witwatersrand and Rahima Moosa Mother and Child Hospital, Johannesburg, South Africa), Ronita Luke (University of Sierra Leone, Freetown), Thomas Misore (Kenya Medical Research Institute, Kisumu), Paul K Mitei (Ministry of Health, Nairobi, Kenya), Alexander M Ibrahim (College of Health and Medical Sciences, Haramaya University, Harar, Ethiopia), Andrew Moultrie University of Witwatersrand and MRC Vaccines and Infectious Diseases Analytics Research Unit, Johannesburg, South Africa), Florence V Murila (Pediatrics and Child Health, University of Nairobi, Kenya), Nellie Myburgh (University of Witwatersrand and MRC Vaccines and Infectious Diseases Analytics Research Unit, Johannesburg, South Africa), Peter Nyamthimba, Richard Oliech, and Richard Omore (Kenya Medical Research Institute, Kisumu), Uma U Onwuchekwa (Centre pour le Développement des Vaccins, Ministère de la Santé, Bamako, Mali), Stian MS Orlien (College of Health and Medical Sciences, Haramaya University, Harar, Ethiopia, and Department of Infectious Disease Epidemiology, London School of Hygiene and Tropical Medicine, United Kingdom), Louis Othieno (Centers for Disease Control and Prevention, Kenya, Kisumu), Peter Otieno (Ethiopian Public Health Institute, Addis Ababa, Ethiopia), Kephas Otieno, Gregory Ouma, and Benard Owuor (Kenya Medical Research Institute, Kisumu), Shahana Parveen (International Center for Diarrhoeal Diseases Research [icddr,b], Dhaka, Bangladesh), Karen L Petersen (University of Witwatersrand, Johannesburg, and Department of Pediatrics and Child Health, Chris Hani Baragwanath Academic Hospital, Soweto, South Africa), Mahbubur Rahman (Institute of Epidemiology, Disease Control, and Research, Dhaka, Bangladesh), Natalia Rakislova (ISGlobal–Hospital Clínic, Unversitat de Barcelona, Spain), Emily A Rogena (Jomo Kenyatta University of Agriculture and Technology, Juja, Kenya), Doh Sanogo (Centre pour le Développement des Vaccins, Ministère de la Santé, Bamako, Mali), Tahmina Shirin (Institute of Epidemiology, Disease Control, and Research, Dhaka, Bangladesh), Diakaridia Sidibe (Centre pour le Développement des Vaccins, Ministère de la Santé, Bamako, Mali), Seydou Sissoko (Sanofi Pasteur, Bamako, Mali), Fatima Solomon (University of Witwatersrand and MRC Vaccines and Infectious Diseases Analytics Research Unit, Johannesburg, South Africa), Gillian Sorour (University of Witwatersrand and Rahima Moosa Mother and Child Hospital, Johannesburg, South Africa), James Sylvester Squire (Ministry of Health and Sanitation, Freetown, Sierra Leone), Peter J Swart (University of Witwatersrand, and National Health Laboratory Service, Johannesburg, South Africa), Fikremelekot Temesgen (Addis Ababa University, Ethiopia), Sharon M Tennant (Center for Vaccine Development and Global Health, University of Maryland School of Medicine, Baltimore), Bukiwe Nana Thwala (University of Witwatersrand and MRC Vaccines and Infectious Diseases Analytics Research Unit, Johannesburg, South Africa), Cheick Bougadari Traore (Centre Hospitalier Universitaire Du Point G, Bamako, Mali), Sithembiso Velaphi (University of Witwatersrand, Johannesburg, and Department of Pediatrics and Child Health, Chris Hani Baragwanath Academic Hospital, Soweto, South Africa), Pio Vitorino (Centro de Investigação em Saúde de Manhiça, Maputo, Mozambique), Jeannette Wadula (University of Witwatersrand, and National Health Laboratory Service, Johannesburg, South Africa), and Melisachew Mulatu Yeshi (Ayder Specialized Comprehensive Hospital, Mekelle University, Mek’ele, Ethiopia).
