# Supplementary material for: Deaths Attributed to Respiratory Syncytial Virus in Young Children in High–Mortality Rate Settings: Report from Child Health and Mortality Prevention Surveillance (CHAMPS)
Source: Clin Infect Dis. 2021 Sep 2;73(Suppl 3):S218–28. doi: 10.1093/cid/ciab509 (PMC8411256; doi:10.1093/cid/ciab509)
Supplement: ciab509_suppl_Supplementary_Tables [file ciab509_suppl_supplementary_tables.docx]

Supplemental Table 1. Definitions of pneumonia and pneumonia caused by RSV used by CHAMPS Determination of Cause of Death (DeCoDe) panels.

| Pneumonia  ICD-10 Codes: J18 (unspecified organism) | |
| --- | --- |
| Level 1 | EITHER Strong histological evidence of pyogenic pneumonia in lung tissue  OR  One of the following laboratory or imaging findings:   - Moderate histological evidence of pneumonia in lung tissue - New infiltrate or pleural effusion on chest radiograph   AND TWO or more of the following clinical signs documented in the medical record:   - Tachypnea (per WHO clinical case definitions, defined as respiratory rate >60/minute in newborns 0-2 months, >50/minute for infants 2-12 months, >40/minute in children 12 months-5 years - Respiratory distress as chest indrawing, grunting or nasal flaring - Abnormal breath sounds (i.e. decreased breath sounds, crackles, crepitations) - Hypoxia, cyanosis or desaturations (oxygen saturation <95%) - Temperature >38.0 (fever) or <36.0 (hypothermia) |
| Level 2 | One of the following:   - No laboratory or imaging data available and ALL of the following documented in the medical record: fever or hypothermia, hypoxia or abnormal breath sounds, and tachypnea or respiratory distress - One of the laboratory or imaging findings above with TWO or more clinical signs of pneumonia above reported by verbal autopsy (difficulty breathing, fast breathing or breathlessness, lower chest wall/ ribs being pulled in or grunting, or fever). |
| Level 3 | Acute febrile illness or hypothermia with tachypnea, respiratory distress, abnormal breath sounds, hypoxia or cyanosis documented in the medical record or reported by verbal autopsy, but not meeting the criteria for Level 1 or Level 2 diagnosis above, OR laboratory evidence of pneumonia but not meeting the criteria for Level 1 or Level 2 diagnosis above. |
| Pneumonia due to respiratory syncytial virus (RSV)  ICD-10 Code: J12.1 | |
| Level 1 | One of the following:   - Strong histological evidence of viral pneumonia and detection of RSV in lung tissue by PCR (TAC) or immunohistochemistry (IHC) - Histological evidence of pneumonia and detection of RSV in lung tissue by PCR or IHC with TWO of the clinical criteria for diagnosis of pneumonia as above, documented in medical record of reported by verbal autopsy - Pneumonia meeting TWO of the clinical criteria for diagnosis of pneumonia as above, as documented in the medical record, with detection of RSV in lung tissue by PCR |
| Level 2 | Pneumonia meeting TWO of the clinical criteria for diagnosis of Level 2 pneumonia above, as reported by verbal autopsy, with detection of RSV in lung tissue by PCR. |
| Level 3 | One of the following:   - Acute febrile illness of hypothermia with tachypnea, respiratory distress, abnormal breath sounds, hypoxia or cyanosis documented in the medial record of reported by verbal autopsy, and detection of RSV in nasopharyngeal/ oropharyngeal swab by PCR - Detection of RSV in the lung tissue by PCR in the absence of sufficient information for Level 1 or Level 2 diagnosis |

Supplemental Table 2. RSV detected in postmortem samples by age at death and CHAMPS site

| **Age at death** | **Number positive for RSV/Number tested (%)** | | | | | | | |
| --- | --- | --- | --- | --- | --- | --- | --- | --- |
|  | **All sites**  **(n=1213)** | **Bangladesh (n=80)** | **Ethiopia (n=28)** | **Kenya (n=281)** | **Mali (n=103)** | **Mozambique (n=125)** | **Sierra Leone (n=99)** | **South Africa (n=497)** |
| <24 hours | 5/289 (1.7) | 2/40 (5.0) | 0/7 (0) | 0/58 (0) | 1/21 (4.8) | 0/50 (0) | 0/11 (0) | 2/102 (2.0) |
| Early Neonate (1 to 6 days) | 2/269 (0.7) | 0/30 (0) | 0/7 (0) | 0/27 (0) | 1/26 (3.8) | 1/17 (5.9) | 0/24 (0) | 0/138 (0) |
| Late Neonate (7 to 27 days) | 6/137 (4.4) | 0/7 (0) | 1/ 2 (50) | 1/17 (5.9) | 2/16 (12.5) | 0/5 (0) | 1/7 (14.3) | 1/83 (1.2) |
| Infant (28 days to <6 months) | 22/184 (12.0) | 0/2 (0) | 1/6 (16.7) | 4/49 (8.2) | 2/14 (14.3) | 0/8 (0) | 1/13 (7.7) | 14/92 (15.2) |
| Infant (6 to <12 months) | 11/99 (11.1) | 0/0 (0) | 0/1 (0) | 2/49 (4.1) | 3/8 (3.8) | 1/11 (9.1) | 0/6 (0) | 5/24 (20.8) |
| Child (12-59 months) | 21/235 (8.9) | 0/1 (0) | 1/5 (4.8) | 11/81 (13.6) | 1/18 (5.6) | 1/34 (2.9) | 0/38 (0) | 7/58 (12.1) |
| TOTAL | 67/1213 (5.5) | 2/80 (2.5) | 3/28 (10.7) | 18/281 (6.4) | 10/103 (9.7) | 3/125 (2.4) | 2/99 (2.0) | 29/497 (5.8) |

Supplemental Table 3. Other causes in the causal pathway to death among children <5 years of age whose deaths were attributed to RSV (n=24), by age group

| Other conditions listed as causes of deaths in addition to RSV | Late neonate (7-27 days)  N=3 | Younger infant (28 days to <6 months)  N=12 | Older infant (6 to <12 months)  N=3 | Child (12 to <60 months)  N=6 |
| --- | --- | --- | --- | --- |
| No other condition | 1 | 3 | - | - |
| Other Lower respiratory infection | 1 | 8 | 3 | 5 |
| Sepsis | 2 | 3 | 2 | 1 |
| Birth Defects | - | 5 | - | 3 |
| Preterm birth complications | 2 | 2 | - | - |
| HIV | - | - | 1 | 2 |
| Tuberculosis | - | - |  | 2 |
| Upper respiratory infection | - | - | 1 | 1 |
| Meningitis | - | 1 | 1 | - |
| Diarrheal diseases | - | - | 1 | - |
| Liver disease | - | - | - | 1 |
| Malnutrition | - | - | 1 | - |
| Motor neuron disease | - | 1 | - | - |
